# A Case of Lupus Erythematosus of the Face and Body, with Superinduced Epitheliomata of the Face

**Published:** 1892-11

**Authors:** Isadore Dyer

**Affiliations:** Lecturer and Clinical Instructor in Dermatology, Medical Department Tulane University and Visiting Dermatologist to Charity Hospital, New Orleans, Etc.


For Daniel's Texas Medical Journal.

### A CASE OF LUPUS ERYTHEMATOSUS OF THE FACE AND BODY, WITH SUPERINDUCED EPITHELIOMATA OF THE FACE.

ISADORE DYER, PH. B., M. D.,

Lecturer and Clinical Instructor in Dermatology, Medical Department Tulane University and Visiting Dermatologist to Charity Hospital, New Orleans, Etc.

S. R., A MAN, active and of good physical development, presents the following clinical history: Aged 54; general condition excellent; no subjective symptoms whatever; is often constipated. On the face, scalp, shoulders and chest an eruption is evident, characterized by discreet lesions, symmetrically arranged, and on both halves of the face and body. Each lesion is red or brown, or pink, and slightly elevated. Each lesion is distinct and has a sharply defined periphery. In size, the lesions vary from a split pea to a five cent nickel. Most of the lesions show, at some part of their periphery, a certain amount of superficial scarring. Many of them present minute tubercles just at the edge of the area affected, and in some, these form the periphery. These characteristics are present in the majority of the lesions which are scattered over the back and chest and on the face. The amount of redness or pigment often varies, and though scaling is the rule, many of the lesions present a smooth surface. There are no subjective sensations, no itching, no burning, etc. On the face, many of the lesions are telangiectic, and some five or six markedly so. These five or six are characteristic. With the sharply elevated peripheral border, firm to the touch, and cartilaginous in appearance, with a center umbilicated and ulcerating, if not scaling, and over and through each coursing a network of blood vessels—the diagnosis of epithelioma is clear. In one or two, these points are hardly yet impressed, but the presence of the other lesions of marked types, points to a necessitous degeneration in the same way. No family history of cancer; no history of tuberculosis. The present eruption began 14 years ago. Patient states that he had a sore on his penis some 20 years ago and was treated for syphilis, though he is sure of no subsequent accidents. Such is the history of the case. The diagnosis was obscure. A history of a possible primary sore, inclined to a diagnosis of syphilis. The eruption on the body suggested a papular and serpiginous syphilide. The history of an epithelioma on top of a late syphilide is by no means uncommon. On the other hand, the patient was free from any adenitis. The general health was unusually good, and the arrangement of the eruption was typical rather of lupus erythematosis than of syphilis, especially of late syphilis. There was nowhere the crescentic grouping so frequent in serpiginous syphilis. The occurrence of the eruption on the body favored the exclusion of lupus erythematosis, for the disease rarely occurs but on the face.

As a sop to Cerberus, antisyphilitic treatment was begun. The patient had been under mixed treatment for several weeks. He was ordered at once, potassium iodide in fifteen drop [?] doses, three times a day. This was ordered increased by 5 drops each day. At the end of the week, the patient showed signs of iodism. He had pains at the angles of his jaws, a certain amount of coryza, and there was a typical papular iodic eruption on his body. The new growths on the face had persisted and progressed, and the ulceration was more marked. The lesions on the face and body were unchanged. The iodide was discontinued. The patient was at once given Thompson's Solution of Phosphorus in 3 drop doses, diluted, after meals. The cancers were curetted thoroughly and the fresh wounds at once cauterized with the Pacquelin point. At this present time, the patient has been under the solution of phosphorus for two weeks. The lesions, generally, are fading. The smaller ones have disappeared, leaving superficial, slightly pigmented scars behind. All the lesions are less elevated, and in only a few are the tubercles at the periphery evident. The wounds which were cauterized, present clean healing surfaces. Two are already cicatrized. The treatment seems to prove the diagnosis, and persistence in the same, increasing the doses of the Thompson's solution and using radical measures locally, will tend to cure the condition and reduce a recurrence to a minimum.

The case is interesting on account of the rarity of such eruption on any part of the body other than the face. It is again interesting from the fact that the development of the epitheliomata should have been induced by the inflammation incident to the lupus erythematosus. Though rare, neither of these conditions is unique, and I report them as interesting in the history of dermatological record.

**Strychnine in Shock, etc.**--In one of the best papers which have appeared lately, "Recent Advances in Therapeutics," H. A. Hare, M. D., in *Lancet-Clinic*, November 5, 1892, Address in Medicine before Mississippi Valley Medical Association, Dr. Hare said:

"I wish to call your attention to the use of strychnine as a remedy for and preventive of surgical shock and anæsthetic collapse, not to speak of its value in opium-poisoning. In these conditions atropine, while very useful, so far as its vaso-motor effects are concerned, does not compare with strychnine either theoretically or practically. To those who habitually employ atropine and morphine injections prior to the use of an anæsthetic, let me recommend the use of strychnine or strychnine and atropine combined. There is one point to be remembered in regard to the use of strychnine in shock or accident, and that is to give it in full doses or leave it alone. Not less than 1-20 grain should be employed hypodermically every half hour in an adult, and, if the condition of shock or respiratory and cardiac failure be marked, one dose of as much as 1-5 grain may be given in this way. Disagreeable effects rarely, if ever, follow, and if they do, will amount to little more than muscular twitching, which can readily be governed by sedatives, for if the drug can stimulate the nervous system sufficiently to cause irritability, it will have pulled the patient out of the 'Slough of Despond,' and he will be able to stand further treatment, should the effect of the strychnine be excessive. Under the conditions spoken of, the man is on the brink of death, and we cannot afford to make haste slowly in dragging him back. A few moments lost and he may be beyond reach, and so far over the edge that human aid cannot draw him back to life."

---

Dr. Reed, of Cincinnati, Secretary-General of the Congress, and chairman of the Committee on Organization, announces that after extended correspondence between himself and Dr. Maragliano, of Genoa, General Secretary of the International Congress, the date of the Rome meeting is finally and definitely set for September 24th, of next year. This gives an interval of sixteen days between the Washington and Rome meetings, during which time an easy trip can be made from the former to the latter city. It is possible that a steamer may be chartered direct from New York to Rome.

